# Case Report: Post-CAR-T Infusion HBV Reactivation in Two Lymphoma Patients Despite Entecavir Preventive Therapy

**DOI:** 10.3389/fimmu.2021.751754

**Published:** 2021-10-08

**Authors:** Yaxian Ma, Li Yang, Yuhan Bao, Yang Yang, Liting Chen, Miao Zheng

**Affiliations:** Department of Hematology, Tongji Hospital, Tongji Medical College, Huazhong University of Science and Technology, Wuhan, China

**Keywords:** hepatitis B virus, reactivation, CAR T, lymphoma, antiviral therapy

## Abstract

Hepatitis B virus (HBV) reactivation is a common complication in chronic or resolved HBV infection patients undergoing immunosuppressive chemotherapy. Furthermore, few articles have been published regarding the risk of HBV reactivation in lymphoma patients receiving chimeric antigen receptor (CAR) T-cell therapy and anti-HBV prophylaxis. Few guidelines or clear optimal strategies are available for managing these patients. Here, we present two cases of patients who underwent CAR-T-cell cocktail therapy with anti-CD19 and anti-CD22 CAR (CAR19/22) T cell for lymphoma. Patients had previous history of HBV infection, and blood tests on initial admission indicated positive results for hepatitis B surface antigen (HBsAg), antibody to hepatitis B core antigen (anti-HBc), and antibody to hepatitis B e antigen (anti-HBe), while serum HBV DNA level was undetectable. Therefore, two patients received entecavir as antiviral prophylactic therapy during their entire treatment. They were diagnosed with HBV reactivation based on positive serum HBV DNA test results, 2 weeks after CAR-T-cell infusion. Liver function assay indicated elevated levels of alanine transaminase (ALT) and aspartate transaminase (AST), combined with increased levels of total bilirubin (TBIL) and direct bilirubin (DBIL). Subsequently, they received anti-HBV treatment with entecavir and tenofovir. As a result, their serum HBV DNA copies and AST/ALT levels returned to normal after 1 week. These cases show that there is a risk of HBV reactivation in lymphoma patients with CAR-T-cell therapy despite entecavir preventive therapy, and combination treatment of entecavir and tenofovir may be an effective treatment option for such patients with HBV reactivation.

## Introduction

Reactivation of hepatitis B virus (HBV), a phenomenon characterized by increased HBV DNA serum values of about 1 log, by the HBV DNA turning positive if previously undetectable in serum or by reverse seroconversion from hepatitis B surface antigen (HBsAg) negative to HBsAg positive, is a well-recognized complication in patients with some cytotoxic chemotherapies (e.g., anthracyclines) or immunochemotherapy for hematologic malignancies (1–4). Guidelines suggest that anti-HBV prophylaxis should be initiated as soon as possible before or, at the latest, simultaneously with starting chemotherapy (4, 5). Once started, antiviral prophylaxis should continue during chemotherapy and for at least 12 months after completion of chemotherapy (4, 5). In patients with lymphoma and resolved HBV infection, entecavir and tenofovir should be considered the drugs for HBV prophylaxis (5).

Among immunotherapies, chimeric antigen receptor-engineered (CAR) T-cell therapy is emerging as a novel and rapidly evolving treatment modality for lymphoma patients (6–8). However, current clinical trials of CAR T-cell therapy have generally excluded lymphoma patients with HBV infection. Thus, the safety and the efficacy of CAR T-cell therapy in patients with lymphoma and HBV infection remain largely unexplored, and reports of HBV reactivation and corresponding clinical solutions are lacking for CAR-T patients, with only a few cases described (2, 9–11). To date, few guidelines or clear optimal strategies are available for the management of HBV reactivation in lymphoma patients undergoing CAR T-cell therapy.

Here, we describe two cases of two patients with lymphoma who experienced HBV reactivation after entecavir preventive therapy and CAR T-cell cocktail therapy with anti-CD19 and anti-CD22 CAR T-cell infusion; HBV hepatitis successfully improved with the combination treatment of entecavir and tenofovir.

## Cases Report

**Figure 1:**
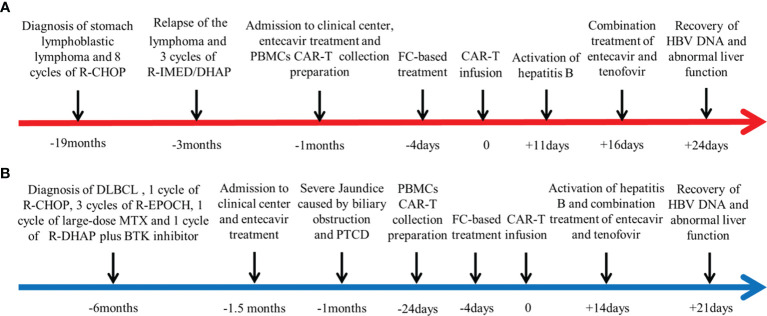
Summaries of the two cases. The first day of chimeric antigen receptor (CAR) T-cell infusion is established as day 0. **(A)** Key clinical course of case 1. **(B)** Key clinical course of case 2.

The key clinical courses of the two patients are summarized in **Figure 1**.

### Case 1

A 55-year-old man was diagnosed with stomach lymphoblastic lymphoma at the IB stage (Ann Arbor staging system) 2 years ago. He received six cycles of standard-dose R-CHOP (rituximab, cyclophosphamide, vincristine, adriamycin, and prednisone). Positron emission tomography–computed tomography (PET-CT) scan showed multiple mass with elevated metabolic activity in the cervical and peritoneal cavities, right clavicula fossa, mediastinal region. Bilateral renal tumor invasion was also detected (Deauville score 5). Progressive disease was considered and rebiopsy of the renal mass was performed. Pathology indicated relapse of the primary disease. The patient then received three cycles of R-IMED/DHAP (rituximab, ifosfamide, methotrexate, etoposide,dexamethasone, cytarabine, and cisplatin) therapy. Posttreatment PET-CT reanalysis suggested partial remission as the metabolic activity of the stomach was significantly repressed (Deauville score 1).

The patient had previous history of HBV infection, and blood test on initial admission indicated positive results for HBsAg, antibody to hepatitis B core antigen (anti-HBc), and antibody to hepatitis B e antigen (anti-HBe), while the serum HBV DNA level was undetectable. His results for human immunodeficiency virus (HIV) and anti-hepatitis C virus (HCV) antibody detection were negative. As for his liver function, serum aspartate aminotransferase (AST) and alanine aminotransferase (ALT) were within the normal range, and no abnormal ultrasound findings were identified suggesting chronic hepatitis. On admission to our clinical center, the patient was required to receive anti-HBV prophylactic treatment with entecavir (0.5 mg/day) during his entire treatment.

**Figure 2:**
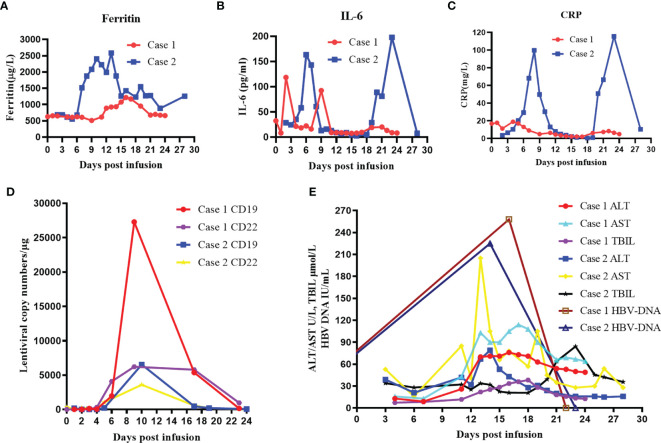
Clinical evolutions after infusion of anti-CD19 and anti-CD22 CAR T-cell cocktail in patients. **(A)** Levels of serum ferritin after CAR T-cell infusion. **(B)** Levels of IL-6 after CAR T-cell infusion. **(C)** Levels of serum CRP after CAR T-cell infusion. **(D)** Lentiviral copy numbers after CAR T-cell infusion. **(E)** Dynamic changes in ALT, AST, TBIL, and HBV DNA levels after CAR T-cell infusion. CAR, chimeric antigen receptor; IL-6, interleukin-6; CRP, C-reactive protein; ALT, alanine transaminase; AST, aspartate transaminase; TBIL, total bilirubin.

Lymphoma of the patient progressed after multiline chemotherapy. In order to control his present conditions, we selected CAR-T cocktail therapy with anti-CD19 and anti-CD22 CAR T-cell infusion. The patient was enrolled in our trial and agreed to receive CAR-T treatment. Informed consent was provided by the patient. Peripheral blood mononuclear cells (PBMCs) were harvested and CAR T cells were constructed and cultured for 14 days by Bio-Rad Corporation (Hercules, CA, USA). Then, standardized FC (fludarabine and cyclophosphamide) regimen was conducted for lymphodepletion (25 mg/m^2^ fludarabine and 20 mg/kg cyclophosphamide on days −4 to −2). Sequential infusion strategy was planned as follows: anti-CD19 CAR-T infusion 2 × 10^6^/kg on days 1 and 3 and anti-CD22 CAR-T infusion 2 × 10^6^/kg on days 0 and 2. The patient gradually developed cytokine release syndrome (grade 1), with peak ferritin level of 952.1 μg/L and interleukin-6 (IL-6) level of 118.50 pg/ml (**Figures 2A–C**). Lentiviral copy surveillance conducted with quantitative reverse transcriptase polymerase chain reaction (qRT-PCR) suggested that CAR T-cell expansion reached a maximum point +9 days post-infusion (copy numbers for CD19 27297 and CD22 6212) (**Figure 2D**).

Although this patient continuously received entecavir (0.5 mg, q.d.) antiviral treatment, he suffered from vomiting and stomach discomfort +11 days post-CAR-T infusion. The liver function assay indicated elevated ALT and AST levels, combined with increased levels of total bilirubin (TBIL) and direct bilirubin (DBIL) (**Figure 2E**). After exclusion of other potential causes of acute liver injury, we examined his HBV DNA copy level, and the results indicated that HBV reactivation was highly possible (+16 days HBV DNA, 2.58 × 10^2^) (**Figure 2E**). Therefore, we quickly implemented combinatory rescuing anti-HBV treatment with entecavir and tenofovir. Reexamination suggested that the patient’s HBV DNA and abnormal liver function recovered on +24 days.

### Case 2

A 46-year old woman was diagnosed with diffuse large B-cell lymphoma 6 months ago (stage IIB). The MRI scan indicated that the lymphoma invaded her liver, pancreas, stomach, and right kidney. She received one cycle of R-CHOP, three cycles of R-EPOCH (rituximab, etoposide phosphate, prednisone, oncovin, cyclophosphamide, and hydroxydaunorubicin), one cycle of large-dose methotrexate (MTX), and one cycle of R-DHAP plus Bruton’s tyrosine kinase (BTK) inhibitor chemotherapy. Posttreatment evaluation indicated that the remaining tumor (13 mm × 10 mm) resided in her lower common bile duct.

This patient had previous history of HBV infection and took entecavir treatment before chemotherapy initiation. On her first admission into our center, serum test indicated that she had positive results of HBsAg, anti-HBe, and anti-HBc. The results for hepatitis C virus (HCV) and HIV antibodies were negative. Her HBV DNA level was undetectable. Her initial liver function test result was quite abnormal as serum AST was 121 U/L and ALT was 190 U/L. Her TBIL level reached 223.9 μmol/L, and the DBIL level was 212.1 μmol/L. We then performed magnetic resonance cholangiopancreatography (MRCP), and the results demonstrated tumor nodule localizing in the lower common bile duct, with notable dilation of the bile duct and pancreatic duct. As her tumor caused serious obstruction, percutaneous transhepatic cholangial drainage (PTCD) was conducted and her liver function quickly recovered.

Because the patient had suffered rapid progression of lymphoma and obstructive jaundice during chemotherapy, chemotherapy resistance was considered. In order to control her pancreatic tumor progression, we performed CAR-T cocktail therapy with anti-CD19 and anti-CD22 CAR-T therapy. Her CAR-T culturing and lymphodepletion chemotherapy were the same as those of case 1. Sequential infusion strategy was planned as follows: anti-CD19 CAR-T infusion 2 × 10^6^/kg on days 1 and 5 and anti-CD22 CAR-T infusion 2 × 10^6^/kg on days 0 and 2. The patient developed mild nausea, vomiting, and fever post-infusion, with peak ferritin level of 2,583 μg/L and IL-6 level of 197.7 pg/ml (**Figures 2A–C**). Lentiviral copy surveillance conducted using qRT-PCR suggested that CAR T-cell expansion reached a maximum point +10 days post-infusion (copy numbers for CD19 6541 and CD22 3602) (**Figure 2D**).

Her liver function remained steady under entecavir treatment until +14 days post-CAR-T infusion. Blood test on +14 days indicated elevated ALT (53 U/L)/AST (66 U/L), with mild elevations of TBIL (22.5 μmol/L) and DBIL (20.7 μmol/L) (**Figure 2E**). After ruling out possibilities of obstruction of the PTCD tube and a retest of HBV copy (+14 days, 2.25 × 10^2^ IU/ml) (**Figure 2E**), reactivation of HBV was suspected. We quickly implemented combinatory rescuing anti-HBV treatment with entecavir and tenofovir. As a result, her HBV DNA copy and her AST/ALT levels returned to normal at +21 days.

## Discussion

In the past two decades, CAR T-cell therapy has been rapidly emerging as a promising novel treatment for hematological malignancies (6–8). CAR T-cell cocktail infusion strategy can reduce tumor antigen escape and improve therapeutic effects (12, 13). Therefore, CAR T-cell cocktail therapy with anti-CD19 and anti-CD22 CAR T-cell infusion was selected for these two patients.

However, as the medical community continues to explore unknown territories that target the immune system to treat various diseases, HBV reactivation remains a vexatious and persistent problem (14). As curative and eradicative therapies for HBV are not currently available, HBV reactivation is a common and potentially fatal complication in patients with previous HBV infection who receive chemotherapies or immunosuppressive therapies (3, 14, 15). HBV is a double-stranded DNA virus that has various genotypes, subtypes, mutants, recombinants, and even quasispecies. Ten genotypes of HBV have been identified, labeled A through J (4). The distribution of these HBV genotypes has obvious geographic-associated features. Infections in East Asia are most commonly HBV genotypes B and C (16). Accumulating lines of evidence have clarified the clinical significance of HBV genotypes and mutants over the past decade (17). HBV genotype is increasingly associated with treatment response, disease severity, and progression (17).

Patients being considered for chemotherapy should be screened for hepatitis B infection with HBsAg, anti-HBe, and anti-HBc and, if needed, subsequent determination of the HBV DNA levels (14, 15, 18, 19). Guidelines suggest that patients with chronic HBV receiving anticancer therapy should receive antiviral prophylactic therapy not only for the entire duration of anticancer therapy but also for at least 12 months post-therapy (4, 5, 15, 18). In those patients with negative HBV DNA levels in serum, periodic determination of the HBV DNA levels and liver enzymes during treatment should be performed (14, 15, 18, 19). With close and regular monitoring of HBV DNA, reactivation events can be captured and the appropriate therapy can be started on time (19).

Patients receiving chemotherapy with hematologic malignancies are at high risk of HBV reactivation. Similarly, CAR T-cell therapy represents a uniquely high-risk group based on the progressive B-cell depletion induced by CAR T cells (15). Our clinical center have reported quickly exacerbated post-CAR-T HBV reactivation cases (9), suggesting that rapid clinical interventions should be made. Case reports of HBV reactivation have been reported after CAR-T therapy in patients with lymphoma and known HBV infection, but the degree of risk has not yet been established (2, 9–11). Taken together, these reports indicate that patients positive for HBsAg may need careful monitoring and require anti-HBV prophylaxis during CAR T-cell therapy (2, 9–11).

The nucleos(t)ide analogues (NAs) of entecavir and tenofovir are considered first-line regimens because of their high potency and low rates of resistance (4, 20). Nucleotide analogue tenofovir has no cross-resistance with nucleoside analogues and can be used to rescue drug resistance during entecavir therapy (21). The American Association for the Study of Liver Diseases (AASLD) guidelines recommend that patients with virologic failure should switch to another first-line agent or add a second drug (4). The consensus of Chinese experts advocates for either a switch to tenofovir monotherapy or a combination entecavir–tenofovir when faced with HBV reactivation in patients treated with entecavir as anti-HBV prophylaxis (22).

In these cases, despite entecavir being chosen as antiviral prophylactic therapy, two lymphoma patients still suffered HBV reactivation after CAR19/22 T-cell cocktail therapy. Reactivation of HBV was successfully treated with the combinatory treatment of entecavir and tenofovir. This prompts us to design a more detailed clinical research to further define the optimal preventive strategy for HBV-infected lymphoma patients receiving CAR T-cell therapy.

In summary, in this study, we demonstrated the risk of HBV reactivation in lymphoma patients receiving CAR T-cell therapy despite entecavir preventive intervention. Our report also indicated the usefulness of entecavir and tenofovir combined therapy for the treatment of HBV reactivation. More data from further clinical studies should be collected to provide clear indications on the following: the risk of HBV reactivation in lymphoma patients receiving CAR T-cell therapy, the duration of anti-HBV prophylaxis and the proper prevention strategy in lymphoma patients receiving CAR T-cell therapy with chronic or resolved HBV infection, and the best drug or combination of drugs for recurrent HBV hepatitis therapy.

## Data Availability Statement

The original contributions presented in the study are included in the article/supplementary material. Further inquiries can be directed to the corresponding author.

## Ethics Statement

The studies involving human participants were reviewed and approved by the Medical Ethics Committee of the Department of Hematology, Tongji Hospital, Tongji Medical College, Huazhong University of Science and Technology. The patients/participants provided written informed consent to participate in this study. Written informed consent was obtained from the individual(s) for the publication of any potentially identifiable images or data included in this article.

## Author Contributions

YM analyzed the data and wrote the first draft of the manuscript. LY managed the patients, collected the clinical data, and wrote a section of the manuscript. YB, YY, LC, and MZ managed the patients. MZ revised the manuscript and was in charge of the final approval of the manuscript. All authors contributed to the article and approved the submitted version.

## Funding

The work was supported by the National Natural Science Foundation of China (no. 81974005) and the Chen Xiao-Ping Foundation for the development of Science and Technology of Hubei Province.

## Conflict of Interest

The authors declare that the research was conducted in the absence of any commercial or financial relationships that could be construed as a potential conflict of interest.

## Publisher’s Note

All claims expressed in this article are solely those of the authors and do not necessarily represent those of their affiliated organizations, or those of the publisher, the editors and the reviewers. Any product that may be evaluated in this article, or claim that may be made by its manufacturer, is not guaranteed or endorsed by the publisher.
